# Supplementary material for: Novel Driver Strength Index highlights important cancer genes in TCGA PanCanAtlas patients
Source: PeerJ. 2022 Aug 11;10:e13860. doi: 10.7717/peerj.13860 (PMC9375969; doi:10.7717/peerj.13860)
Supplement: Supplemental Information 2 [file peerj-10-13860-s002.zip › COHORTS/genes plots/detailed/2021_8_16_14_9_distribution_events_detailed_32.pdf]

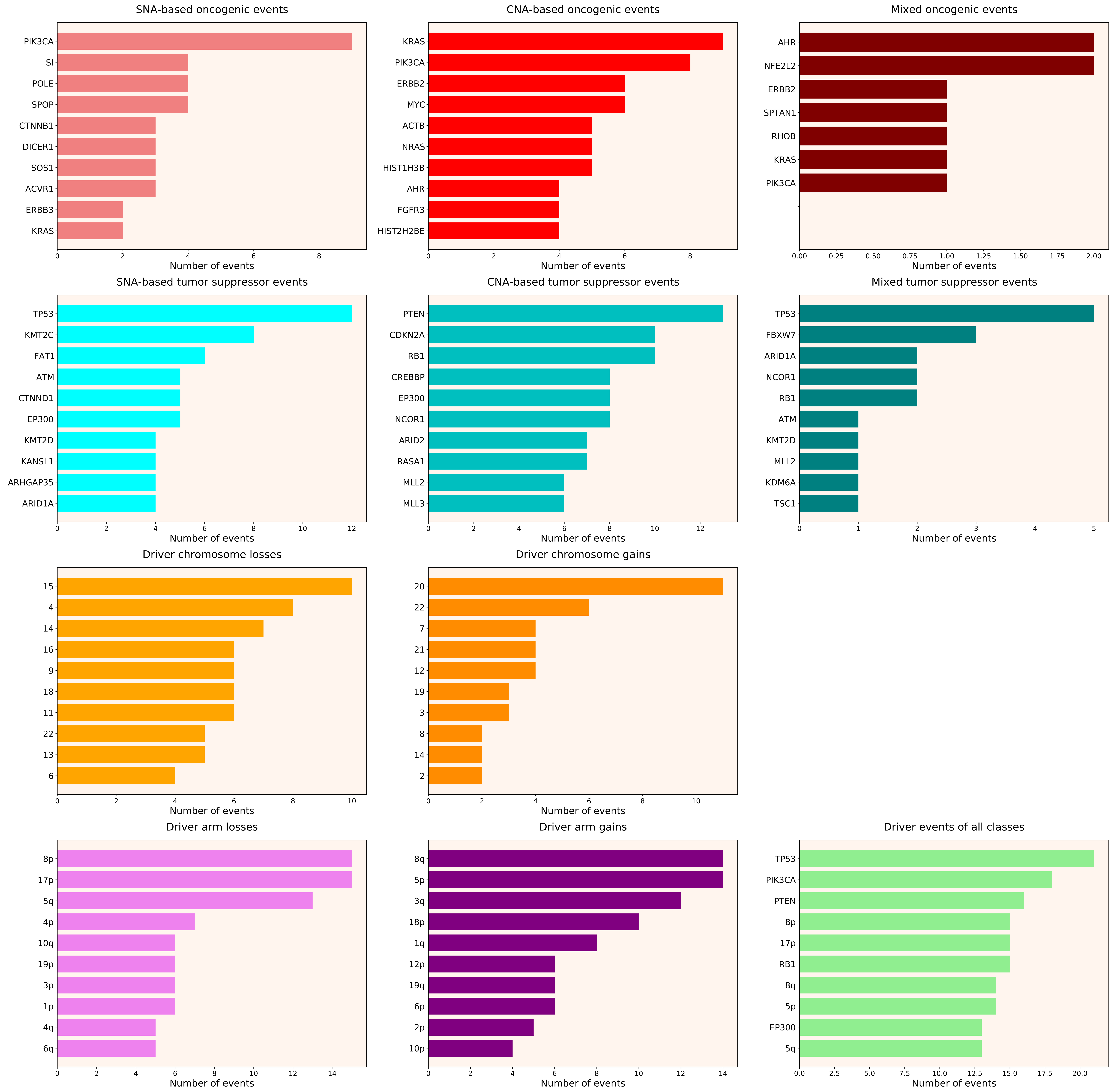

SNA-based oncogenic events
PIK3CA
SI
POLE
SPOP
CTNNB1
DICER1
SOS1
ACVR1
ERBB3
KRAS
0 2 4 6 8
Number of events
CNA-based oncogenic events
KRAS
PIK3CA
ERBB2
MYC
ACTB
NRAS
HIST1H3B
AHR
FGFR3
HIST2H2BE
0 2 4 6 8
Number of events
Mixed oncogenic events
AHR
NFE2L2
ERBB2
SPTAN1
RHOB
KRAS
PIK3CA
0.00 0.25 0.50 0.75 1.00 1.25 1.50 1.75 2.00
Number of events
SNA-based tumor suppressor events
TP53
KMT2C
FAT1
ATM
CTNND1
EP300
KMT2D
KANSL1
ARHGAP35
ARID1A
0 2 4 6 8 10 12
Number of events
CNA-based tumor suppressor events
PTEN
CDKN2A
RB1
CREBBP
EP300
NCOR1
ARID2
RASA1
MLL2
MLL3
0 2 4 6 8 10 12
Number of events
Mixed tumor suppressor events
TP53
FBXW7
ARID1A
NCOR1
RB1
ATM
KMT2D
MLL2
KDM6A
TSC1
0 1 2 3 4 5
Number of events
Driver chromosome losses
15
4
14
16
9
18
11
22
13
6
0 2 4 6 8 10
Number of events
Driver chromosome gains
20
22
7
21
12
19
3
8
14
2
0 2 4 6 8 10
Number of events
Driver arm losses
8p
17p
5q
4p
10q
19p
3p
1p
4q
6q
0 2 4 6 8 10 12 14
Number of events
Driver arm gains
8q
5p
3q
18p
1q
12p
19q
6p
2p
10p
0 2 4 6 8 10 12 14
Number of events
Driver events of all classes
TP53
PIK3CA
PTEN
8p
17p
RB1
8q
5p
EP300
5q
0.0 2.5 5.0 7.5 10.0 12.5 15.0 17.5 20.0
Number of events
